# Increases in subsistence farming due to land reform have negligible impact on bird communities in Zimbabwe

**DOI:** 10.1002/ece3.8612

**Published:** 2022-02-12

**Authors:** Stephen Pringle, Ngoni Chiweshe, Martin Dallimer

**Affiliations:** ^1^ Sustainability Research Institute School of Earth and Environment University of Leeds Leeds UK; ^2^ CIRAD Zimbabwe, c/o IUCN Zimbabwe Harare Zimbabwe

**Keywords:** biodiversity conservation, DPCoA, functional redundancy, functional traits, land‐use change, species' richness

## Abstract

Habitat alterations resulting from land‐use change are major drivers of global biodiversity losses. In Africa, these threats are especially severe. For instance, demand to convert land into agricultural uses is leading to increasing areas of drylands in southern and central Africa being transformed for agriculture. In Zimbabwe, a land reform programme provided an opportunity to study the biodiversity response to abrupt habitat modification in part of a 91,000 ha dryland area of semi‐natural savannah used since 1930 for low‐level cattle ranching. Small‐scale subsistence farms were created during 2001–2002 in 65,000 ha of this area, with ranching continuing in the remaining unchanged area. We measured the compositions of bird communities in farmed and ranched land over 8 years, commencing one decade after subsistence farms were established. Over the study period, repeated counts were made along the same 45 transects to assess species' population changes that may have resulted from trait‐filtering responses to habitat disturbance. In 2012, avian species' richness was substantially higher (+8.8%) in the farmland bird community than in the unmodified ranched area. Temporal trends over the study period showed increased species' richness in the ranched area (+12.3%) and farmland (+6.8%). There were increased abundances in birds of most sizes, and in all feeding guilds. New species did not add new functional traits, and no species with distinctive traits were lost in either area. As a result, species' diversity reduced, and functional redundancy increased by 6.8% in ranched land. By 2020, two decades after part of the ranched savannah was converted into farmland, the compositions of the two bird communities had both changed and became more similar. The broadly benign impact on birds of land conversion into subsistence farms is attributed to the relatively low level of agricultural activity in the farmland and the large regional pool of nonspecialist bird species.

## Graphical abstract

A land reform programme provided a unique opportunity to study the biodiversity response to abrupt habitat modification in an extensive dryland area of Zimbabwe. We measured the changing compositions of bird communities in transformed and untransformed land over an 8‐year period, commencing one decade after subsistence farms were established. Temporal trends showed a broadly benign impact, with increased abundances across all feeding guilds, and in species of virtually all sizes, accompanied by reduced species diversity and increased functional redundancy.

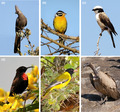

## 1 INTRODUCTION

Habitat modification and land‐use change, primarily due to rising human populations and demand for food, are major contributors to biodiversity loss (De Camargo & Currie, 2015; Murphy & Romanuk, 2014). Around a third of all terrestrial land is now used for food production (Diaz et al., 2020) and species' losses have increased dramatically in recent decades. African ecosystems are particularly exposed to threats posed by land‐use change, as the continent is home to a human population that is growing at an estimated annual rate of 2.7% (UN, 2019). The combined pressures of population growth, increased food demand, and land tenure reform are expected to lead to widespread human‐driven habitat modification. Small‐scale subsistence farming is expected to increase following conversion of marginal drylands, an extensive biome covering nearly 3 million km^2^ in central and southern Africa (Shorrocks, 2007). Drylands, characterised by low and erratic rainfall, are especially vulnerable to biodiversity loss, but the impact of land change on biodiversity in this biome has received little attention (Garcia‐Vega & Newbold, 2020).

Intensified land‐use and habitat degradation often results in more‐specialised species being replaced by generalists, leading to functional homogenisation in changed communities with fewer distinct functional traits (Clavel et al., 2011), and altered ecosystem functioning (Díaz et al., 2007). But this view that land‐use intensification inevitably gives rise to species' loss, leading to a loss of functional traits' diversity and ecosystem function, is not unchallenged. Mayfield et al. (2010) have argued that research does not support a cascade loss for all natural systems, and that community responses depend upon the intensity and spatial extent of disturbance, species' traits and pool size, the level of functional redundancy, and environmental filtering effects. There is also evidence that the impact on biodiversity of abrupt land change may not be permanent. Across 5,563 global sites of varying sizes and levels of disturbance (PREDICTS database; Hudson et al., 2017), local species' richness and abundance in eight taxonomic groups were reduced within 5 years of abrupt land change, but local biodiversity recovered to levels comparable with unchanged sites within a decade (Jung et al., 2019).

The Zimbabwe Fast‐Track Land Reform Programme (FTLRP), introduced in 2000 to address historical patterns of inequitable land distribution, resulted in large parts of the country being transformed for subsistence farming. Between 2000 and 2007, over 8 million hectares were converted into farmland by new resettled farmers, many of whom lacked experience, resources, support, and access to training (DeGeorges & Reilly, 2007; Moyo & Matondi, 2008). In one area of Matabeleland, 650 km^2^ of dryland savannah were transformed into farmland during 2001–2002. This savannah landscape of poor soils, used for low‐level ranching but otherwise largely unmodified and uninhabited for at least eight decades before 2001, was representative of the ‘natural’ habitat of Matabeleland. The transition into farmland provided an opportunity to study the impact of abrupt land‐use change on biodiversity by assessing the trajectory followed by the avian community in the impacted area. We commenced our study in 2012, counting birds along transects in land modified for farming and also in adjacent unmodified ranched savannah. We used our comparative data for the farmed and ranched area bird communities in 2012 to assess the divergent trend followed by farmland birds over the decade following habitat modification. Then, by using 2012 data as a baseline, our repeated counts of identical transects until 2020 enabled us to measure the extent to which different species and functional groups were affected by habitat change. We hypothesised that: (a) avian taxonomic composition and functional diversity of the farmed and ranched area communities would increasingly diverge, with species' richness and functional redundancy increasing in farmland as new species with similar traits moved in; and (b) species' richness and diversity in the ranched area would remain broadly stable, with this area increasingly becoming a refuge for larger birds and those with specialist traits.

## 2 METHODS

### 2.1 Study area and survey methods

**FIGURE 1:**
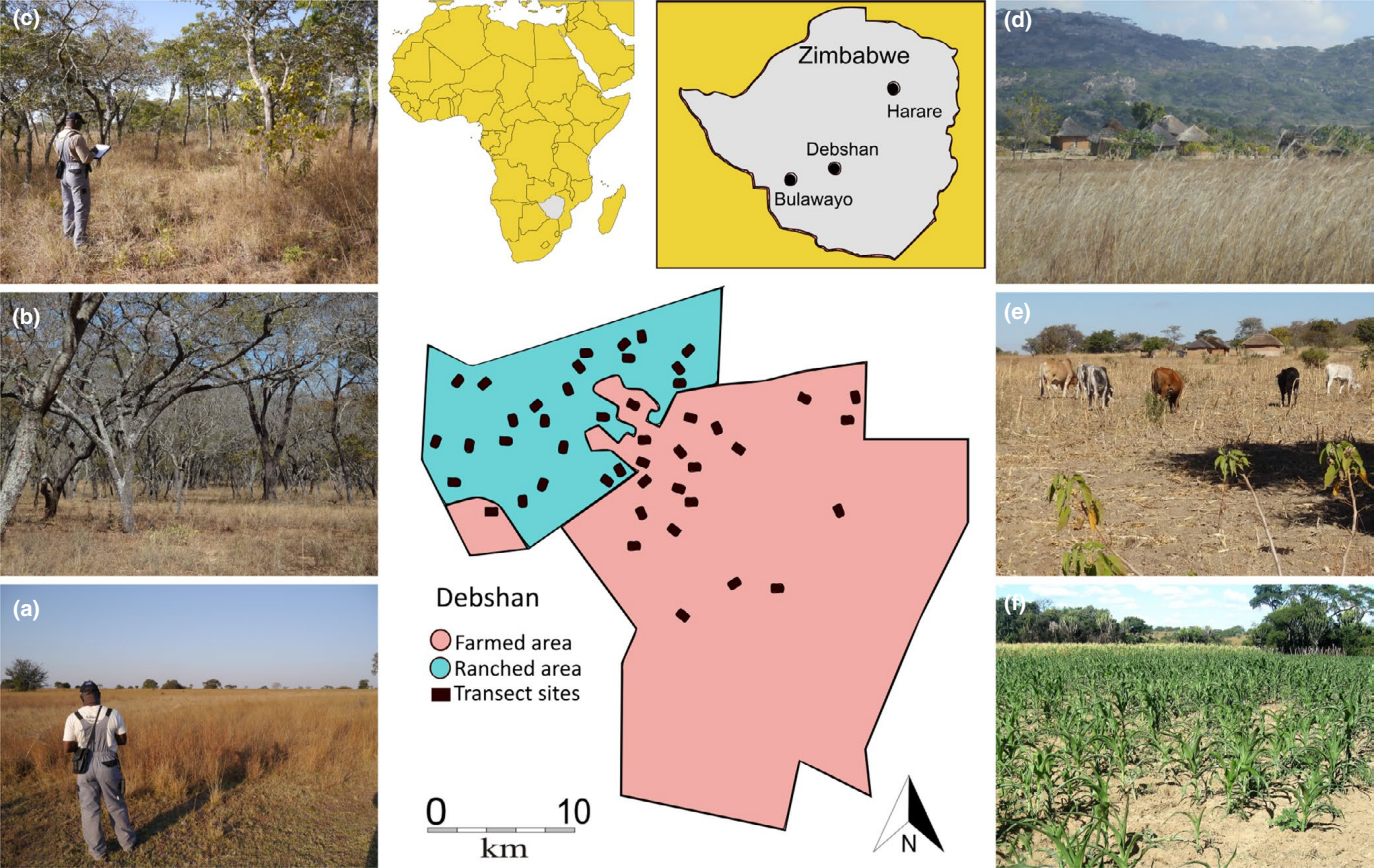
Location of the study area in Zimbabwe showing the transect survey sites in farmed and ranched areas. The three main habitats, photographed in winter, are (a) open grassland, (b) miombo woodland, and (c) acacia woodland. Homesteads in the farmed area have small adjacent fields that provide winter fodder (d‐e) and summer crops such as maize (f). Photos: Stephen Pringle (a‐d); Martin Dallimer (e); Ngoni Chiweshe (f)

The study area in south‐central Zimbabwe is a 91,000‐ha mosaic of dryland savannah comprising open grassland interspersed with wooded areas of acacia (e.g., *Acacia* spp., *Terminalia* spp.) and miombo (e.g., *Brachystegia* spp., *Julbernardia* spp.) trees varying in height from 3–10 m (Figure 1). This area (centred on 29°34′E, 20°04′S), located on poor Kalahari sands, has long been regarded as unsuitable for commercial agricultural crops, and the entire site was formerly used for low‐level cattle ranching. Apart from this activity, these extensive lands were relatively undisturbed as an informally protected area within the private De Beers Shangani Estate (Debshan) since 1930. The FTLRP legislation resulted in a 65,000‐ha demarcated section of Debshan being allocated for resettlement farms. During 2001–2002 approximately 3,000 families were moved to 5‐ha plots (in total 15,000‐ha) distributed across the resettlement area, where they built homesteads, grazed livestock, and established small fields for crops during the summer rainy season. We estimate that, at this time, about 45% (29,000‐ha) of the total land demarcated for resettlement was nominally suitable for subsistence crop cultivation, with the remaining area comprising rocky and hilly outcrops, woodland, and small dams. The main crop grown is maize, with smaller quantities of sorghum, finger millet, various pulses (cow peas, ground nuts, round nuts, beans), pumpkins, water melons and cotton. During 2002–2015, a steady influx of new settlers more than doubled the human population in the farmed area (our estimate; there are no official census data). This resulted in all potentially suitable habitat in the resettled farmed area being converted for homesteads, livestock grazing, and crop production. Since 2015, this trend has plateaued and the population has stabilised as a result of drought and movement of younger people back to cities.

We define two land‐use types for our study: “farmed,” the newly resettled lands used for subsistence farming; and “ranched,” the remaining untransformed land, which continues, essentially unchanged, in private ownership with low‐level cattle ranching (about one head of cattle per 6‐ha).

Our analysis of Google Earth images from 2011 showed that farmed and ranched lands both contained similar, evenly distributed, mosaics of three fragmented habitat types: open grasslands (48% by area), miombo woodlands 30%, and acacia woodlands 22%. These proportions enabled us to define the number of transects needed in each area and habitat type in order for our surveys to be representative of the entire study site. We did not aim to assess changes in bird communities within each habitat type. A set of linear transects defined by GPS coordinates and with random start points and orientations were identified within each habitat (Figure 1). In total, 45 sites were surveyed: 23 ranched (acacia *n* = 5, miombo *n* = 7, open *n* = 11) and 22 farmed (acacia *n* = 5, miombo *n* = 6, open *n* = 11). These descriptions indicate the dominant habitat in that transect; the proportions of each transect‐type match the habitat percentages in each land‐use area. To avoid pseudo‐replication, transects in ranched and farmed areas of the same habitat type were spaced well apart. Distances (mean, SD, closest) between sites were acacia (16.1; 3.2; 3.5) km; miombo (13.3; 1.8; 3.4) km; open (11.2; 1.1; 3.6) km.

Surveys were undertaken during the winters (June–July) of 2012, 2014, 2016, 2018, and 2020 by the same observer team (lead observer NC; recorders MD, SP), along identical transects, and using the same methods. Two 600 m transects, parallel and spaced 300 m apart, were walked at constant slow speed shortly after sunrise (from 05:30), or before sunset (from 16:00), on clear, dry days. Two sites were counted on each day, with sites randomly assigned to morning or afternoon and located as far apart as possible in different habitat types. Birds were only recorded visually, and data collected were distance to the bird(s) using a Leica LRF1200 rangefinder, the number of individuals, and the angle of deviation from the transect. All birds over‐flying the transects were disregarded, and great care was taken to avoid double counting. Indications of human activities and the presence of game animals observed at all distances from transects were also recorded: numbers of people, buildings, livestock, dogs, game animals, presence of standing water, and evidence of tree cutting.

### 2.2 Data analyses: Input data, species' richness, and abundances

We ran EstimateS 9.1.0 software (Colwell, 2013) on individual‐based count data to evaluate sampling adequacy and calculate Chao1 estimators of species' richness (SR). Differences in species' richness between land‐uses were assessed in terms of effect size (ES), calculated as: ES = Absolute (SR_ranched_ – SR_farmed_)/pooled population standard deviation (Smart et al., 2009). We highlight ES values >1.0 as indicators of potentially important ecological changes (Smart et al., 2009).

We used Distance 7.1 software (Thomas et al., 2010), applied separately to transect counts for each year and land‐use, to calculate species' abundances corrected for variable probabilities of detection. Records of birds sighted at distances >100 m from transect lines were discarded. Conventional Distance Sampling mode was used, with 2 modeling options: half normal functions with Cosine series expansion and uniform functions with simple polynomial series expansion (Buckland et al., 2001). The most parsimonious model solution was chosen using Akaike's Information Criterion (Buckland et al., 2001). In the analyses, every species was grouped into one of 11 classes of perceived detectability (“prominence,” Table A1), by which we categorized the conspicuousness and behavior of that species based on our extensive field experience in African ornithology. This method allowed counts of all species, including those rarely observed, to be adjusted for variable detectability and inclusion in subsequent analyses of abundances and population densities (Pringle et al., 2019).

We used counts during 2012–2020 to estimate temporal trends in individual species and in bird communities in ranched and farmed areas. To do so, we used a two‐step process involving the R‐based software packages “rtrim” and “BRC indicators” (R Core Team, 2019). These methods are used to assess trends in annual abundance indices from national bird counts in European countries (PECBMS, 2021). In the first step (rtrim), we used species' abundances, corrected for detection probabilities, to calculate population indices and standard errors adjusted for the effects of overdispersion and serial correlation between years (Pannekoek & van Strien, 2005). We used these outputs in a log‐linear Poisson regression (BRC indicators) to calculate the slopes and 95% CIs of the population trends. This method applies Monte Carlo procedures to account for sampling errors and generate confidence intervals for multi‐species indicators (MSIs) and trends in MSIs. In our model, we ran 5,000 simulations, using 2012 as the base year with MSI value set at 1 and standard error zero. The trend in each species, or group of species, is determined by calculating the multiplicative trend, which reflects changes in terms of the average percentage change per year. The overall population trend is then converted into a trend category based on the multiplicative trend and its 95% confidence interval. There are six categories, ranging from “strong increase” to “steep decline” (Table A2; Soldaat et al., 2017).

### 2.3 Data analyses: Species' traits, diversity, and functional analyses

We compiled a database of traits for every species from standard references (Brown et al., 1982; Fry & Keith, 2004; Fry et al., 1988, 2000; Keith et al., 1992; Urban et al., 1986, 1997). Our database included nine traits per species: five measurements of morphology (average adult body mass; lengths of wing, tail, bill, and tarsus), bill shape (16 categories), primary feeding guild (frugivore, granivore, insectivore, nectarivore, omnivore, and predator), nest type (six categories), and average clutch size (Table A3). These traits were chosen to reflect distinctive aspects of species as well as relating to resource usage that drives ecosystem functions (Şekercioğlu, 2006). Body metrics reflect resource consumption (mass), foraging mode and behavior (bill and tarsus), and flight range for resource access and dispersal (wing and tail). Bill shape and primary feeding guilds are relevant in terms of ecosystem services, population control, resource removal and nutrient recycling. Nest type reflects the role of birds as ecosystem engineers, e.g., in providing structures that host other organisms, or in modifying trees or soil by excavating cavity nests. Temporal changes in the avian communities recorded in ranched and farmed areas were evaluated by combining this traits database with species' abundances in each year.

We follow Pavoine (2020) in defining diversity in the two land‐use areas: species' diversity is the number of species present (= species' richness), weighted by the abundance of each species; phylogenetic beta diversity is the difference between communities in positions of species on the abundance‐weighted phylogenetic trees. An R‐based software package, “div,” and associated functions “divparam” and “abgevodivparam” (Pavoine, 2020; R Core Team, 2019) were used to measure species' diversity and phylogenetic beta diversity, together with changes in these indices during 2012–2020. These functions include a parameter (*q*) that controls the relative weighting of rare and abundant species, which aids in interpreting trends. Functional redundancy, measured in terms of distances between species in the functional traits dendrogram and weighted by species' abundances, was calculated using the “uniqueness” function. This technique quantifies redundancy by comparing the observed community to one in which traits of all species are maximally dissimilar (Pavoine, 2020).

To analyze temporal trends in the phylogenetic compositions of communities in the two land‐use areas, we used a version of double principal coordinate analysis (DPCoA; Pavoine et al., 2013) to include the effects of two crossed factors. The crossed‐DPCoA method, available within the package “adiv,” uses ordination techniques within a mathematical space in which species' abundances, their traits dissimilarities, and two factors (in our case, land‐use type and year) are represented by a set of points. The method allows the interacting effects of the two factors to be decomposed, i.e., the effect of land‐use type is separated from the year of survey with regard to variations in phylogenetic composition (Pavoine, 2020).

## 3 RESULTS

Some indications of changes in the farmed area during 2012–2020 are given by our indirect measures of human impact (Table 1). The number of people encountered during our transect counts is not systematic or representative of overall human population size and pressures. However, when compared with transect counts in the ranched area, there are 10–20 times as many people present in farmland. The number of buildings seen from the transects virtually doubled over 8 years in farmland, suggesting an increasing human population. New buildings in the ranched area relate to modified grazing methods, which have also impacted the numbers of cattle seen on ranched transects. Livestock trends in farmland are unclear; after increasing rapidly during 2012–2016, numbers have declined, possibly reflecting drought conditions following low summer rainfall in 2018–2019 (Figure S1). Drought conditions, combined with disease, may have been responsible for the reduced number of dogs. Game animals are now largely restricted to the ranched area.

**TABLE 1:** Aspects of human impact recorded in transect counts during 2012–2020

		2012	2014	2016	2018	2020
People	Ranched	10	5	21	29	14
	Farmed	180	228	285	211	197
Buildings	Ranched	7	7	18	20	27
	Farmed	436	588	554	504	790
Water present	Ranched	3	3	4	10	5
	Farmed	6	10	9	12	7
Livestock	Ranched	454	376	241	10	439
	Farmed	406	609	927	634	461
Dogs	Ranched	1	1	2	1	1
	Farmed	50	78	31	38	7
Game animals	Ranched	271	221	303	336	191
	Farmed	30	6	2	3	9
Transects with cut trees	Ranched	1	1	3	5	3
	Farmed	20	22	22	21	22

Note

Data show numbers seen from transect lines at all observable distances, i.e., not limited to 100 m.

For each year, habitat, and land‐use type, numbers of species recorded approached asymptotes, suggesting that only a few uncommon species were overlooked in each survey set. In 2012, species' richness was 8.8% higher in farmland than in the ranched area, and it continued to be higher throughout the study period, with an effect size >1 in all years except 2014 (Table 2). However, the ranched area species' richness also increased by 12.3% during 2012–2020 as new species colonized that area.

**TABLE 2:** Throughout the study period, more bird species were recorded in farmland, compared with ranched land

	2012	2014	2016	2018	2020
Ranched transects SR	98.1	117.5	97.4	107.1	110.2
SD	4.80	5.22	1.68	11.60	2.25
Farmed transects SR	106.8	119.9	117.0	123.7	114.9
SD	1.89	2.58	4.90	3.14	4.53
Effect size	**2.38**	0.58	**5.40**	**1.94**	**1.34**

Note

Biennial count data from identical winter transects during 2012–2020 were used to calculate avian species' richness (SR) and standard deviation (SD), based on Chao 1 estimates. Differences in species' richness between ranched (552 ha) and farmed (528 ha) transects in the same year were assessed in terms of effect size (ES), calculated as: ES = Absolute (SR_ranched_ – SR_farmed_)/Pooled population standard deviation. We highlight ES values >1.0 (in bold) as indicators of potentially important ecological differences between communities.

**FIGURE 2:**
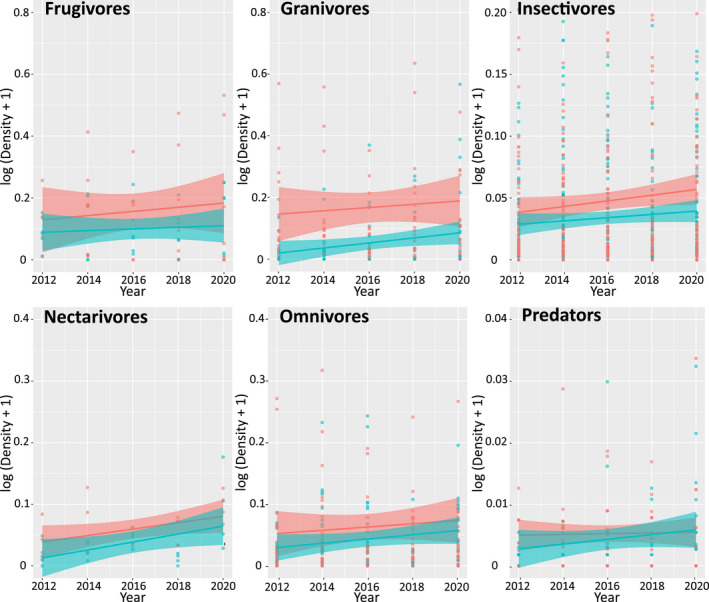
Birds in virtually all primary feeding guilds and land‐use areas were increasingly abundant over the study period (farmland trend: predators uncertain). Data points (red: farm; blue: ranch) are log‐transformed densities of every species recorded during biennial counts of identical winter transects from 2012 to 2020. Species' counts are corrected for detection probability; each species is then assigned to its primary feeding guild. Lines are linear regressions, with shading indicating 95% CIs. The significance of these trends is assessed using packages “rtrim” and “BRC indicators,” which calculate population indices and standard errors adjusted for the effects of overdispersion and serial correlation between years (Table 3)

**FIGURE 3:**
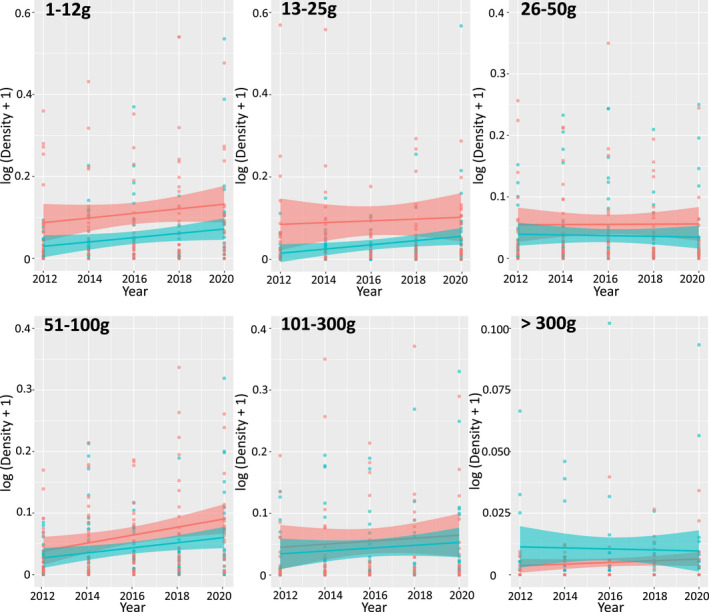
Birds in most mass ranges and land‐use areas were increasingly abundant over the study period (ranched area trends: 26–50 g stable; >300 g uncertain). Data points (red: farm; blue: ranch) are log‐transformed densities of every species recorded during biennial counts of identical winter transects from 2012 to 2020. Species' counts are corrected for detection probability; each species is then assigned to a mass range according to their average adult body mass. Lines are linear regressions, with shading indicating 95% CIs. The significance of these trends is assessed using packages “rtrim” and “BRC indicators,” which calculate population indices and standard errors adjusted for the effects of overdispersion and serial correlation between years (Table 3)

**FIGURE 4:**
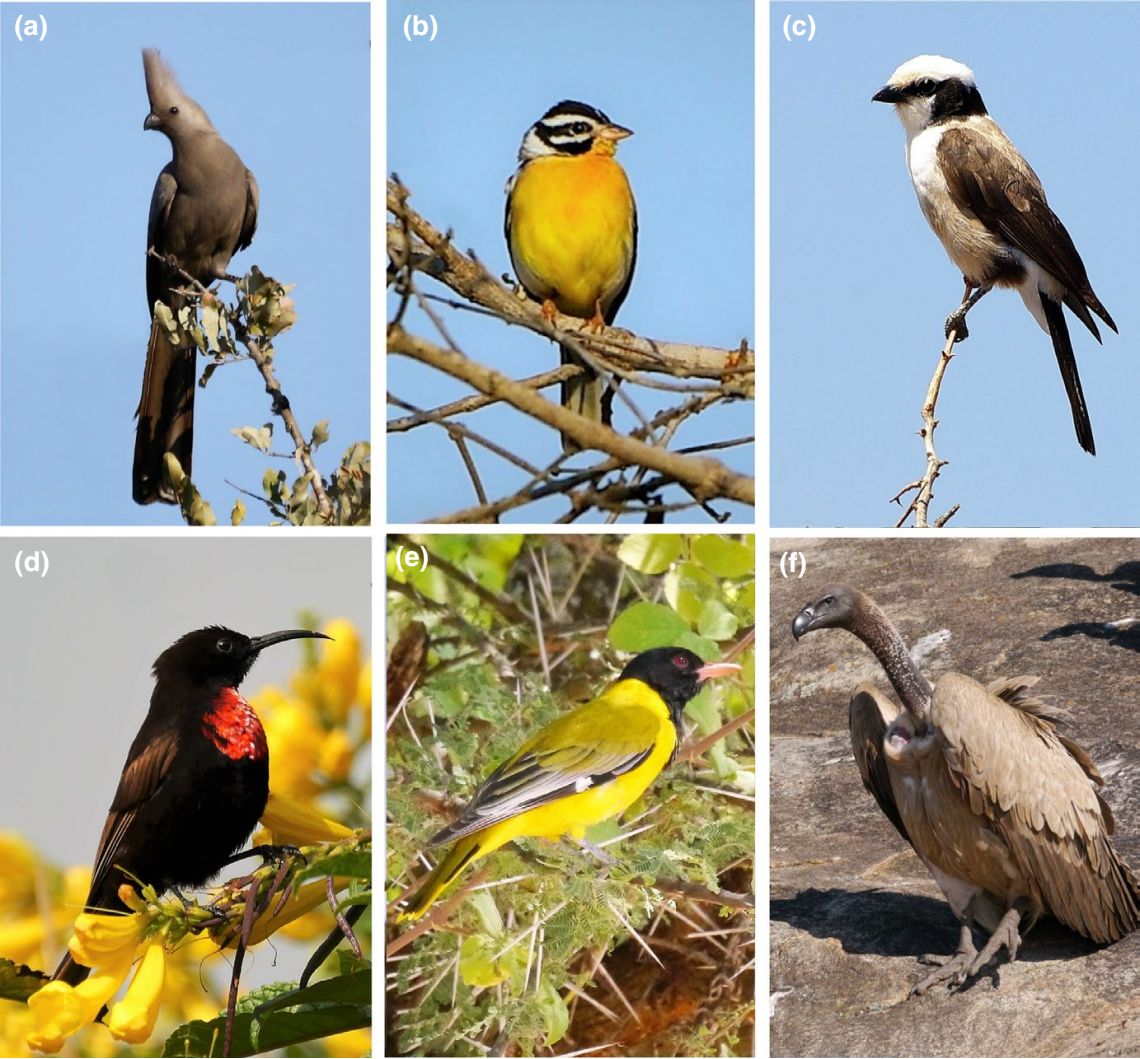
Abundances of many species in different feeding guilds increased strongly in farmed and ranched areas during 2012–2020, including (a) Grey Go‐away‐bird (frugivore); (b) Golden‐breasted Bunting (granivore); (c) Southern White‐crowned Shrike (insectivore); (d) Scarlet‐chested Sunbird (nectarivore); and (e) Black‐headed Oriole (omnivore). Raptor abundances were stable; a higher density in the ranched area largely reflects White‐backed Vultures (f) roosting in the vicinity of nest sites. Photos: Stephen Pringle

With the possible exception of predators in farmland, abundances of birds in all primary feeding guilds, and in both land‐use areas, increased during 2012–2020 (Figure 2). When analyzed by species' average body mass, abundances also increased in most mass ranges (Figure 3). The MSI technique, which corrects for overdispersion and serial correlation between years, confirmed significant moderate or strong increases in abundance of most categories of birds (Table 3; Table A2). These increases occurred in a large number of individual species across a range of feeding guilds (Figure 4), and few species showed moderate or steep declines in either area during 2012–2020 (Table A4). The analyses were restricted to species with total numbers >50 recorded in both areas across all surveys. However, even with this cut‐off level, many uncommon species are included, as the limit equates to 5 individuals/year recorded across all transects in each land‐use area.

**TABLE 3:** Population trends of species grouped by primary feeding guild and by average body mass

		Community trend during 2012–2020			
		Ranched area		Farmed area	
		Trend ± SE	Category	Trend ± SE	Category
Guild	Frugivore	1.151 ± 0.018	Strong increase	1.188 ± 0.016	Strong increase
	Granivore	1.267 ± 0.020	Strong increase	1.179 ± 0.009	Strong increase
	Insectivore	1.048 ± 0.010	Moderate increase	1.099 ± 0.009	Strong increase
	Nectarivore	1.434 ± 0.051	Strong increase	1.198 ± 0.034	Strong increase
	Omnivore	1.198 ± 0.016	Strong increase	1.117 ± 0.012	Strong increase
	Predator	1.207 ± 0.065	Strong increase	1.098 ± 0.055	Uncertain
	All guilds	1.162 ± 0.007	Strong increase	1.143 ± 0.005	Strong increase
Mass	1–12 g	1.316 ± 0.017	Strong increase	1.122 ± 0.009	Strong increase
	13–25 g	1.118 ± 0.040	Moderate increase	1.119 ± 0.010	Strong increase
	26–50 g	1.021 ± 0.014	Stable	1.050 ± 0.012	Moderate increase
	51–100 g	1.190 ± 0.016	Strong increase	1.201 ± 0.013	Strong increase
	101–300 g	1.151 ± 0.017	Strong increase	1.125 ± 0.015	Strong increase
	>300 g	0.988 ± 0.200	Uncertain	1.243 ± 0.075	Strong increase
	All masses	1.162 ± 0.007	Strong increase	1.143 ± 0.005	Strong increase

Note

The trends are generated using the multispecies indicator function “msi” in the BRC indicators package (Soldaat et al., 2017). The significance of trends and their classification are as defined in Table A2.

**FIGURE 5:**
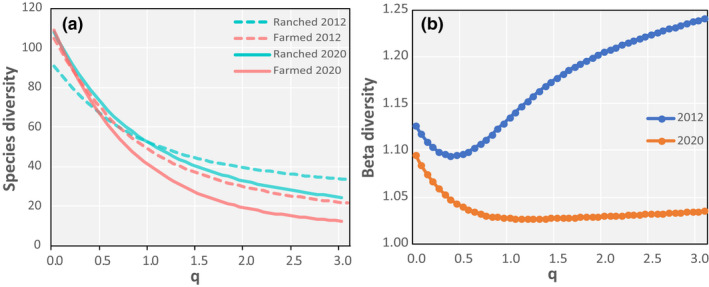
(a) Avian species' diversity curves differed between farmed and ranched areas, and shifted between 2012 and 2020. The parameter q controls the sensitivity of species' diversity to abundance‐weighting of each species. At *q* = 0, species' abundances are disregarded and reflect presence/absence, thus the y‐intercept is the observed species' richness for the community. In effect, at *q* = 0, rare species are given higher weighting than common species. For *q* > 0, species' diversity increasingly accounts for abundance until at *q* = 3, abundant species are given high weight and rare species low weight; (b) phylogenetic beta diversity between ranched and farmed bird communities decreased from 2012 (blue) to 2020 (brown). As in (a), parameter q controls the sensitivity of this diversity index to the abundance weighting of each species. In 2012, phylogenetic differences between birds in different land‐use types were highest for more abundant species, whereas differences reduced and were confined to rarer species (low q values) in 2020

Species' diversity curves, modulated by abundance weighting, show marked differences between bird communities according to land use and year (Figure 5a). In 2012, there was higher species' richness (*q* = 0, representing presence/absence) in farmed areas (105 vs 91 species), but higher species' diversity in the ranched area for *q* > 0.7 as abundance weighing increased. In contrast, the species' diversity curves for 2020 show almost identical species' richness (*q* = 0, 109 vs 108 species). Compared with 2012, the lower diversity values in 2020 at *q* = 3 indicates that common species were increasingly dominant in both areas. However, even with these species given high weighting, in 2020 the bird community in the ranched area continued to have higher species' diversity than in farmland. These trends are reflected in the phylogenetic beta diversity curves, which show that the traits‐based dissimilarity between ranched and farmed area bird communities was lower in 2020 than in 2012 for all values of *q* (Figure 5b).

**FIGURE 6:**
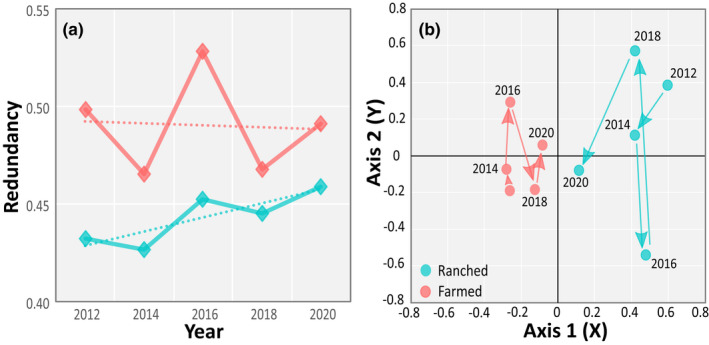
Bird communities in farmed and ranched areas became increasingly similar between 2012 and 2020. (a) Functional redundancy increased in the ranched area (blue) bird community, approaching the level of farmland birds (red). Redundancy values are calculated using distances between species in the functional traits dendrogram, weighted by species' abundances. Dotted lines are linear regressions, which show unchanged functional redundancy during 2012–2020 in the farmland bird community (Slope = −0.0011 ± 0.0093 with *R* ^2^ = .005; *F*(1, 3) = 0.014; *p* = .914), but a significant redundancy increase among those species present in the ranched area (Slope = 0.0080 ± 0.0024 with *R* ^2^ = .782; *F* (1,3) = 10.740; *p* = .047). (b) Differences in the composition of bird communities decreased over time (as indicated by converging count year arrow sequences) and were smallest in 2020. Over the period 2012–2020, the greatest changes (arrow length and direction) occurred in the ranched area community. The communities in each year are represented by points derived from nonmetric ordination, which distils the main patterns of species' richness, abundance, and traits present in each land‐use onto two principal axes. Increasingly similar communities result in more closely clustered points

Linear regressions show unchanged functional redundancy during 2012–2020 in the farmland bird community (Slope = −0.0011 ± 0.0093 with *R* ^2^ = .005; *F*(1,3) = 0.014; *p* = .914), but a significant redundancy increase among those species present in the ranched area (Slope = 0.0080 ± 0.0024 with *R* ^2^ = .782; *F*(1,3) = 10.740; *p* = .047) (Figure 6a).

The first stage of crossed‐DPCoA analysis of species' abundances and functional traits, with land‐use type (A) and year (B) as factors, generated an ordination plot showing the positions of communities around the first two axes (Figure 6b). The principal (X) and secondary (Y) axes expressed 40% and 32%, respectively, of the variance in the position of the levels of factor A. Along the X‐axis, communities in ranched areas are clearly separated on the positive side of the origin from those in farmland on the negative side. The sequences of transect counts in ranched and farmed areas show a converging pattern during 2012–2020, with the greatest changes occurring in the ranched area community. The close proximity of the 2020 points indicates that the two communities were the most similar in that year.

**FIGURE 7:**
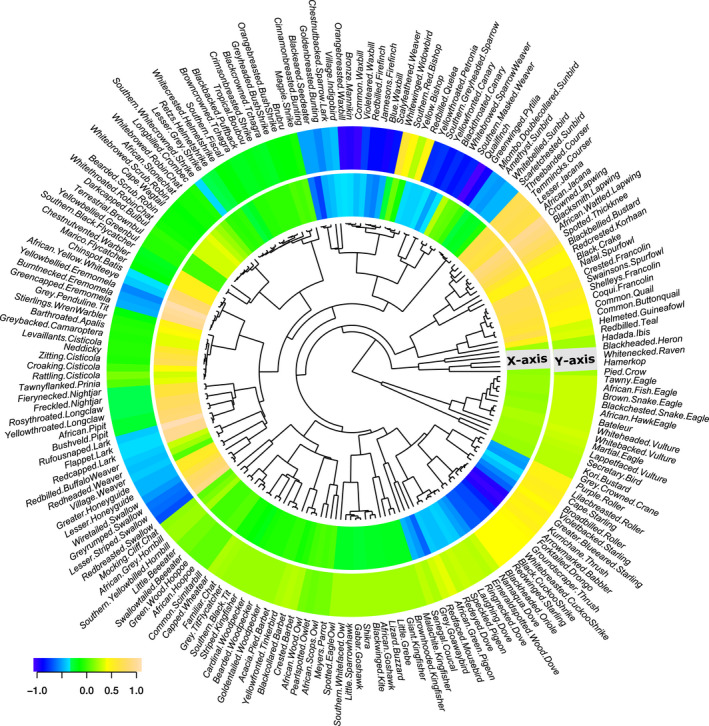
There were proportionately more small granivores and large insectivores in farmland in 2012–2016, while the ranched area held more small insectivores and ground‐dwelling birds. However, this pattern changed from 2016 as new species colonized the ranched area. This DPCoA analysis shows trends in the phylogenetic composition of bird communities in each land‐use area, with the central dendrogram showing functional traits' dissimilarities between species. Interpretation of this figure is in two stages. In the first stage, consider the (primary) X‐axis of Figure 6b, which shows that all bird communities in the ranched area lie on the positive side of that axis, with all farmland communities on the negative side. In this figure, the color‐coded scale (+1 to −1) relates to the ± axes values in Figure 6b. The colored ring labeled “X‐axis” displays the relative proportion of each species in each area. Species forming a higher proportion of the ranched area community are shaded yellow‐brown, indicating distance (increasing proportion) along the positive X‐axis. In the same way, shades of blue (negative X‐axis) indicate a higher proportion in farmland, while green shading indicates equal proportions in communities of both land‐use areas. In the second stage, consider the (secondary) Y‐axis of Figure 6b and again apply the colour‐coding convention. The pattern of point distribution here is more complex and harder to interpret as the survey years for ranched and farmed area communities are not clearly separated relative to the Y‐axis origin. However, points furthest from the Y‐axis origin carry the greatest weight and dominate trends reflected in this figures, i.e., changes in the ranched area community (positive in 2018, negative in 2016). This suggests that, in these years, some of the trends observed on the X‐axis were changing, or even reversing. For example, the proportion of small, predominantly granivorous species (e.g., waxbills, weavers, and canaries) strongly increased in the ranched area in 2016. This area also gained more rollers, starlings, and thrushes in 2018

Trends in the proportions of individual species in each land‐use area during 2012–2020 are shown in Figure 7. The central dendrogram shows functional traits dissimilarities between species. The differences between bird communities were mostly due to the higher proportion of small granivores (e.g., waxbills, canaries, and doves) and larger insectivores (e.g., rollers, starlings, and thrushes) in farmland in 2012–2016, during which time the ranched area held higher proportions of small insectivores (e.g., cisticolas, eremomelas) and ground‐dwelling birds such as lapwings and spurfowl. In 2016 and 2018, some of the earlier trends in species' abundances were changing, or even reversing. For example, in 2016, small granivorous birds (e.g., waxbills, weavers, and canaries) strongly increased in abundance in the ranched area. The ranched area also gained more rollers, starlings, and thrushes in 2018.

## 4 DISCUSSION

For many decades prior to 2001, the entire study area was uninhabited savannah used for low‐level cattle ranching. In 2001–2002, abrupt human settlement, accompanied by building of homesteads and commencement of subsistence farming, resulted in widespread habitat modification in a part of this area. This resulted in a matrix of subsistence farms, interspersed with areas of uncropped grassland and woodland patches, replacing the former contiguous savannah. Although the resettled farming households are now well established, their reliance on farming in unproductive shallow sandy soils leads to a tenuous existence. Droughts and socioeconomic instability have meant that many younger people leave the farms to work in urban areas, thereby limiting growth in the community (pers. obs.).

The immediate impact of rapid land conversion during 2001–2002 on bird species' richness and abundance in the farmed part of our study area is unknown. However, our 2012 results show that, by then, these indices were similar to (or exceeded) levels in ranched land. This is consistent with the >10‐year biodiversity recovery period from abrupt land change estimated by Jung et al. (2019). Our further surveys to 2020 show that, after a time‐lag well in excess of 10 years from abrupt disruption, the bird community in farmed land restructured in a way that increased species' richness with loss of diversity. In the adjacent ranched land, a similar trajectory was followed, but with an additional time lag. Although some other studies of land conversion in Africa (e.g., Baudron et al., 2019; Coetzee & Chown, 2016; Marcacci et al., 2020; Mulwa et al., 2012; Norfolk et al., 2017) have identified benefits for certain bird groups, our results suggest an overall benign impact on the entire bird community in this specific case. The increased species' richness that we recorded in the ranched area was unexpected, as the habitat in this area has remained unchanged.

Bird population densities increased considerably over the survey period, with moderate to strong increases across a wide range of species in all feeding guilds. Some guilds (e.g., granivores) are expected to benefit from land conversion to agriculture, but it is surprising that, in our study area, abundances increased in all guilds, and in all areas. Abundances appear to be unrelated to average adult body mass, with stability or increasing populations in all mass ranges, with the possible exception of ranched area birds with mass >300 g. Although the reasons for these increasing abundances are unclear, nationwide surveys in grassland, savannah, and woodland habitats in neighboring Botswana recorded a strong increase in bird populations during 2010–2015. In Botswana, 49% of recorded species showed significant increases, and common species fared best outside protected areas (Wotton et al., 2017). A similar pattern is observed in our data, which shows increased abundances in 56%–64% of those species recorded in sufficient numbers to permit analysis (Table A4).

The differing profiles of species' diversity curves for bird populations indicate that, although species' richness was higher in farmland in 2012, species' diversity was higher in the ranched area when abundances were taken into account. By 2020, species' diversity profiles had shifted as some species that were only in farmland in 2012 spread into the ranched area, increasing richness in that area, but leaving it unchanged in farmland. The changed composition of the populations is also reflected in the phylogenetic beta diversity curves for 2012 and 2020, which show marked differences in the dissimilarity profiles between the ranched and farmed communities. In 2012, phylogenetic differences between birds in different land‐use types were highest for more abundant species, whereas differences reduced and were confined to rarer species in 2020.

These diversity trends are confirmed by changes in other indices. Trends in functional redundancy, a measure of the abundance of species with similar traits, differed according to land use. In the farmed area, it was relatively stable, while increasing redundancy was recorded in the ranched area bird community. Communities impacted by land‐use change may follow a number of different trajectories as they adapt and restructure following disturbance (Mayfield et al., 2010). In our study, the trends should reflect the environmental filtering effects of subsistence farming on the bird community that was initially present in the unmodified dryland savannah. At the start of our study in 2012, species' richness and functional redundancy were higher in farmland than in the ranched area, suggesting that additional species from the regional species' pool had colonized farmland after land‐use change in 2002, but had added few new traits. This pattern is expected in tropical areas, where species' pools are large (Mayfield et al., 2010). During 2012–2020, further new species colonizing the farmland added no new traits as functional redundancy remained largely unchanged. In contrast, in the untransformed ranched land, functional redundancy increased during 2012–2020. If species' richness in this area had declined or remained constant, this would have suggested that some species with diverse traits were lost, then partly or fully replaced by an influx of new species with similar traits. However, ranched area species' richness increased, and no loss of bird species was apparent over the survey period. It appears that the composition of the bird communities in the two land‐use areas started to converge, with new species becoming increasingly abundant, initially in farmland, and later in the ranched land, but contributing few new functional traits.

Our DPCoA analysis reveals the major changes that occurred in the phylogenetic composition of bird communities during our 8‐year study. Throughout the study period, about 50% of species maintained broadly similar proportions of the communities present in each land‐use area. Some differences we recorded in functional groups (e.g., a higher proportion of granivores in farmland) were to be expected on the basis of other research in Africa (e.g., Gove et al., 2013; Greve et al., 2011; Sinclair et al., 2002). The availability of suitable food in the vicinity of crops and homesteads is likely to have benefitted over 25 species of doves, pigeons, seedeaters, waxbills, and buntings in the farmland. Several of these species (e.g., Jameson's Firefinch, Common Waxbill) were not recorded in the ranched area in 2012 and appear to have been early colonizers of the farmland. Other trends in farmland, such as proportionately more medium‐sized frugivores, insectivores, and omnivores (e.g., rollers, starlings, thrushes, go‐away birds), suggest that they too benefitted from habitat change. The trends in the above functional groups in farmland led to lower proportions of some other functional groups such as ground‐dwelling birds (e.g., lapwings, spurfowl) compared with the ranched area community. By 2016 and 2018, some earlier trends in phylogenetic composition were changing, or even reversing. For example, in 2016, small granivorous birds (e.g., waxbills, weavers, and canaries) strongly increased in the ranched area. The ranched area also gained more rollers, starlings, and thrushes in 2018. The converging sequence of points in the ordination plot provides further evidence of the two bird communities becoming more similar with increased time since the habitat was transformed in the farmed area.

All of the bird species in this study have a wide distribution in southern Africa. Of the 187 species we recorded, all except nine are classed as Least Concern (IUCN, 2021). The birds of conservation concern include three vulture species and three eagles. Of the vulture species in the study area, White‐backed Vultures *Gyps africanus* (Critically Endangered) have established a growing breeding colony in the ranched area (but outside our transects). Although numbers were small, the Secretarybird *Sagittarius serpentarius* (Endangered) was more often recorded in the farmed area, rather than ranched land. In South Africa, this species has adapted to transformed areas in South Africa, but declined inside the protected Kruger National Park (Hofmeyr et al., 2014). Grey Crowned Cranes *Balearica regulorum* (Endangered) occurred only in the farmed area, and Kori Bustards *Ardeotis kori* (Near Threatened) were restricted to ranched land; numbers of both species were low.

This study supports growing evidence that, where interspersed with intact natural habitat, subsistence farming in Africa can support an abundant and richly diverse avian community. Recent research findings from Kenya (Norfolk et al., 2017) and Ethiopia (Baudron et al., 2019; Marcacci et al., 2020) suggest that, for taxa such as birds, a multifunctional landscape that includes small‐scale agriculture can play an important role in biodiversity conservation. Common factors that link these studies are the presence of a wide range of habitat‐generalist species, and the heterogeneous habitat mosaics in which low‐level farming activities are embedded. Harsh environmental conditions in this newly farmed area of Zimbabwe placed natural constraints on farming activities and human impact over the past two decades, and the modified landscape retained much of the original habitat within the agricultural matrix. Our study provides a unique insight into the initial impact of, and subsequent recovery from, an abrupt land‐use change event in an understudied dryland biome.

## CONFLICT OF INTEREST

The authors have no conflicts of interest to declare.

## AUTHOR CONTRIBUTIONS

**Stephen Pringle:** Data curation (supporting); formal analysis (lead); investigation (supporting); methodology (equal); writing – original draft (lead); writing – review and editing (equal). **Ngoni Chiweshe:** Conceptualization (equal); data curation (lead); investigation (lead); methodology (equal); writing – original draft (supporting); writing – review and editing (equal). **Martin Dallimer:** Conceptualization (equal); data curation (supporting); investigation (supporting); methodology (equal); writing – original draft (supporting); writing – review and editing (equal).

## Figures and Tables

**FIGURE A1:**
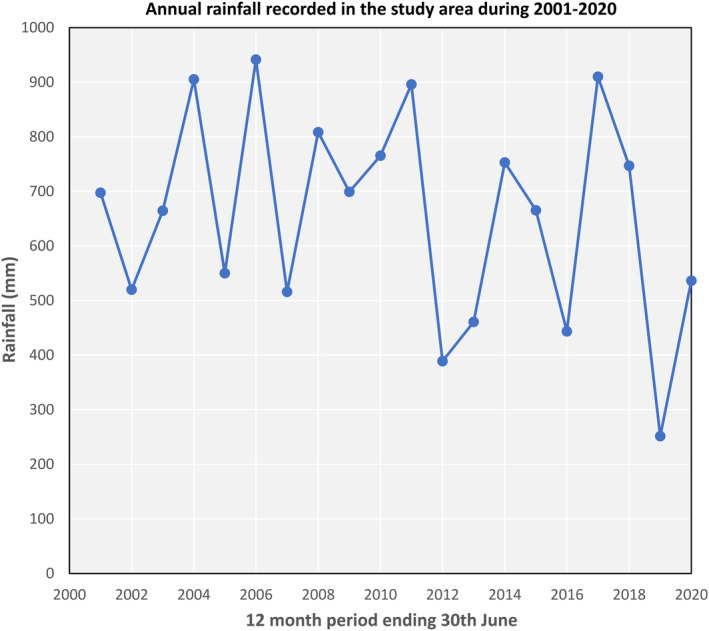
Annual rainfall recorded in the study area during 2001–2020

## Data Availability

Data used in the analyses are accessible from the Research Data Leeds Repository (http://archive.researchdata.leeds.ac.uk/) under citation: Pringle (2022).

## APPENDIX A

**TABLE A1:** Categories and definitions of prominence codes assigned to bird species recorded across all habitats in farmed and ranched areas of the study site

Code	Description	Examples
cry	Cryptic or secretive	Nightjars, owls, bitterns, coursers, thick‐knees, quails, cuckooshrikes
fli	Aerial feeders	Swifts, swallows, martins, bee‐eaters
flo	Flocking birds	Queleas, weavers, waxbills, mannikins, bishops, widowbirds, whydahs
lbb	Large bush birds	Hornbills, turacos, pigeons, large doves, rollers, coucals
lgr	Large ground dwellers	Lapwings, guineafowl, spurfowl, francolins
lob	Large birds; birds of prey	Bustards, herons, crows, ravens, hamerkops, vultures, eagles, buzzards, kestrels, falcons
mbb	Medium bush birds	Drongos, small doves, thrushes, starlings, cuckoos, orioles, honeyguides, babblers
sbb	Small bush birds	Robins, chats, bulbuls, shrikes, seedeaters, canaries, sparrows, flycatchers
sgr	Small ground dwellers	Larks, pipits, wagtails, longclaws, buntings, wheatears, sparrow larks, hoopoes
tbb	Tiny bush birds	Tits, eremomelas, camaropteras, white‐eyes, warblers, crombecs, prinias, cisticolas, sunbirds
tre	Tree specialists	Woodpeckers, barbets, parrots, kingfishers, wood hoopoes, scimitarbills
**Code**	**Description**	**Rationale**
cry	Cryptic or secretive	Birds (mostly cryptically coloured) which are unlikely to be seen unless disturbed; lurking birds in all habitats.
fli	Aerial feeders	Aerial‐feeding insectivores; quite vocal, and often flying repeated circuits.
flo	Flocking birds	Often feed together in flocks comprising one or more of these species; flocking behaviour draws attention.
lbb	Large bush birds	Large birds (135 g < m < 270 g) that tend to feed (in/from) and perch in bushes or trees. Hard to overlook in acacia/miombo.
lgr	Large ground dwellers	Large birds (all m > 150 g) that reside and feed exclusively on the ground. Can be cryptic depending upon habitat.
lob	Large birds; birds of prey	Very large size and/or behaviour (e.g., prominent perching, aerial circling, vocal) give high visibility.
mbb	Medium bush birds	Medium birds (40 g < m < 134 g, and all cuckoos) that often feed (in/from) or perch in bush/trees. Less visible than large bush birds.
sbb	Small bush birds	Small birds (mostly 20 g < m < 39 g, and all shrikes) that tend to feed (in/from) and perch in bushes or trees. Can join bird parties.
sgr	Small ground dwellers	Small birds (all m < 55 g) that reside and feed exclusively on the ground. Can be cryptic depending upon habitat.
tbb	Tiny bush birds	Tiny birds (mostly m < 20 g) that tend to feed (in/from) and perch in bushes or trees. Can be hard to see, but often in bird parties.
tre	Tree specialists	Birds that reside and feed exclusively in/from trees. Nest in tree holes. Generally vocal, colourful.

**TABLE A2:** Categories of trends in populations based on the slope and 95% CI output of software packages “rtrim” and “BRC indicators” (Soldaat et al., 2017)

Trend category	Trend slope (S)	95% CI lower limit (L)	95% CI upper limit (U)
Strong increase	S > 1.05	L > 1.05	None
Moderate increase	1.00 < S ≤ 1.05	1.00 < L < 1.05	None
Stable	Any	0.95 ≤ L	U ≤ 1.05
Uncertain	Any	Either 0.95 > L	or U > 1.05
Moderate decline	0.95 ≤ S < 1.0	None	0.95 < U < 1.00
Steep decline	S < 0.95	None	U < 0.95

**TABLE A3:** List of bird species recorded across all transects during 2012–2020 showing primary feeding guilds, morphological measurements, bill type, nest type, and average clutch size

Standard IOC Name	Scientific Name	Guild	Mass	Wing	Tail	Culmen	Tarsus	Bill	Nest	Clutch
Acacia Pied Barbet	Tricholaema leucomelas	f	30	82	49	20	19	ser	hol	2.9
African Fish Eagle	Haliaeetus vocifer	p	2,820	559	252	41	85	hoo	plt	2.0
African Goshawk	Accipiter tachiro	p	356	230	198	17	63	hoo	plt	2.5
African Green Pigeon	Treron calvus	f	231	171	99	13	22	sle	plt	1.5
African Grey Hornbill	Tockus nasutus	m	208	215	192	88	36	cas	hol	4.0
African Hawk‐Eagle	Hieraaetus spilogaster	p	1,420	440	272	31	95	hoo	plt	1.6
African Hoopoe	Upupa africana	i	53	137	92	49	19	dec	hol	3.4
African Jacana	Actophilornis africanus	i	182	156	45	52	65	pro	gnd	3.6
African Pipit	Anthus cinnamomeus	i	27	87	64	14	26	sli	gnd	2.7
African Scops Owl	Otus senegalensis	i	69	137	65	11	22	hoo	hol	2.7
African Stonechat	Saxicola torquatus	i	15	72	52	16	23	sli	cup	3.2
African Wattled Lapwing	Vanellus senegallus	i	224	232	99	34	85	pro	gnd	3.6
African Wood Owl	Strix woodfordii	p	299	249	153	30	46	hoo	hol	2.0
African Yellow White‐eye	Zosterops senegalensis	i	11	59	40	10	15	sho	cup	2.8
Amethyst Sunbird	Chalcomitra amethystina	n	11	64	41	24	16	dec	ovl	1.8
Arrow‐marked Babbler	Turdoides jardineii	i	72	110	108	24	32	sle	cup	2.8
Bar‐throated Apalis	Apalis thoracica	i	11	52	55	13	20	sho	ovl	2.7
Bateleur	Terathopius ecaudatus	p	2,242	527	109	36	73	hoo	plt	1.0
Bearded Scrub Robin	Cercotrichas quadrivirgata	i	26	80	73	18	26	sli	cup	2.8
Bearded Woodpecker	Dendropicos namaquus	i	83	132	67	31	19	chi	hol	3.0
Black Crake	Amaurornis flavirostra	m	94	103	42	25	40	pro	gnd	4.0
Black Cuckoo‐Shrike	Campephaga flava	i	34	104	100	15	19	sle	cup	1.9
Black‐backed Puffback	Dryoscopus cubla	i	27	80	71	19	22	hoo	cup	2.7
Black‐bellied Bustard	Lissotis melanogaster	i	1,966	353	186	44	131	pro	gnd	1.5
Black‐chested Snake Eagle	Circaetus pectoralis	p	1,962	510	272	34	87	hoo	plt	1.0
Black‐collared Barbet	Lybius torquatus	m	59	92	57	23	21	ser	hol	3.3
Black‐crowned Tchagra	Tchagra senegalus	i	51	86	101	23	28	hoo	cup	2.5
Black‐eared Seedeater	Serinus mennelli	g	15	81	52	11	13	con	cup	3.0
Black‐headed Heron	Ardea melanocephala	p	1,078	401	157	100	136	poi	plt	2.8
Black‐headed Oriole	Oriolus larvatus	m	65	137	97	28	22	sle	cup	2.4
Blacksmith Lapwing	Vanellus armatus	i	156	211	88	28	73	pro	gnd	3.4
Black‐throated Canary	Serinus atrogularis	m	11	71	43	9	12	con	cup	3.0
Black‐winged Kite	Elanus caeruleus	p	248	272	122	17	36	hoo	plt	3.5
Blue Waxbill	Uraeginthus angolensis	g	11	52	54	10	14	con	ovl	3.5
Bronze Mannikin	Lonchura cucullata	g	9	49	30	10	14	con	ovl	2.7
Broad‐billed Roller	Eurystomus glaucurus	i	105	176	98	22	17	sle	hol	4.9
Brown Snake Eagle	Circaetus cinereus	p	2,048	514	270	43	100	hoo	plt	1.0
Brown‐crowned Tchagra	Tchagra australis	i	33	76	94	18	24	hoo	cup	2.4
Brown‐hooded Kingfisher	Halcyon albiventris	p	64	107	66	49	16	poi	hol	3.7
Brubru	Nilaus afer	i	24	84	57	16	22	hoo	cup	2.0
Burnt‐necked Eremomela	Eremomela usticollis	i	9	55	43	12	20	sho	cup	2.6
Bushveld Pipit	Anthus caffer	i	16	72	53	11	17	sli	gnd	2.5
Cape Starling	Lamprotornis nitens	i	88	132	90	23	34	sle	hol	2.8
Cape Wagtail	Motacilla capensis	i	21	82	84	14	23	sli	cup	2.8
Capped Wheatear	Oenanthe pileata	i	33	94	59	15	31	sli	hol	3.0
Cardinal Woodpecker	Dendropicos fuscescens	i	31	94	47	19	16	chi	hol	2.4
Chestnut‐backed Sparrow Lark	Eremopterix leucotis	g	13	83	46	11	16	con	gnd	1.9
Chestnut‐vented Warbler	Sylvia subcoerulea	i	15	66	68	12	21	sho	cup	2.5
Chinspot Batis	Batis molitor	i	12	60	47	13	18	sho	cup	1.7
Cinnamon‐breasted Bunting	Emberiza tahapisi	g	14	77	60	10	16	con	cup	3.0
Common Buttonquail	Turnix sylvaticus	m	45	81	32	11	19	sto	gnd	6.6
Common Quail	Coturnix coturnix	m	96	105	36	13	24	sto	gnd	6.6
Common Scimitarbill	Rhinopomastus cyanomelas	i	37	108	125	42	19	dec	hol	2.7
Common Waxbill	Estrilda astrild	g	8	49	56	9	15	con	ovl	4.9
Coqui Francolin	Peliperdix coqui	m	261	132	75	22	37	sto	gnd	5.0
Crested Barbet	Trachyphonus vaillantii	m	71	102	86	23	26	ser	hol	2.9
Crested Francolin	Dendroperdix sephaena	m	342	151	95	22	44	sto	gnd	6.5
Crimson‐breasted Shrike	Laniarius atrococcineus	i	48	99	100	23	32	hoo	cup	2.7
Croaking Cisticola	Cisticola natalensis	i	21	66	59	14	28	sho	ovl	3.3
Crowned Lapwing	Vanellus coronatus	i	155	202	91	31	68	pro	gnd	2.7
Dark‐capped Bulbul	Pycnonotus barbatus	f	39	97	87	17	21	sli	cup	2.6
Emerald‐spotted Wood Dove	Turtur chalcospilos	g	64	111	84	18	18	sle	plt	2.0
Fiery‐necked Nightjar	Caprimulgus pectoralis	i	55	161	120	12	16	wid	gnd	3.1
Familiar Chat	Oenanthe familiaris	i	21	85	62	16	24	sli	hol	1.9
Flappet Lark	Mirafra rufocinnamomea	i	26	81	55	14	22	con	gnd	2.2
Fork‐tailed Drongo	Dicrurus adsimilis	i	51	134	119	21	22	sle	cup	2.8
Freckled Nightjar	Caprimulgus tristigma	i	79	190	132	13	19	wid	gnd	2.0
Gabar Goshawk	Micronisus gabar	p	155	195	163	13	45	hoo	plt	2.3
Giant Kingfisher	Megaceryle maxima	p	364	206	117	87	16	poi	hol	3.5
Golden‐breasted Bunting	Emberiza flaviventris	g	18	82	69	13	17	con	cup	2.4
Golden‐tailed Woodpecker	Campethera abingoni	i	68	118	65	27	17	chi	hol	2.9
Greater Blue‐eared Starling	Lamprotornis chalybaeus	f	86	131	90	19	32	sle	hol	3.5
Greater Honeyguide	Indicator indicator	i	48	109	70	14	15	sto	par	3.0
Green Wood Hoopoe	Phoeniculus purpureus	i	71	154	236	51	22	dec	hol	3.0
Green‐capped Eremomela	Eremomela scotops	i	9	57	47	11	18	sho	cup	2.5
Green‐winged Pytilia	Pytilia melba	m	15	59	49	13	15	con	ovl	3.8
Grey Crowned Crane	Balearica regulorum	m	3772	565	239	62	207	pro	gnd	2.6
Grey Go‐away‐bird	Corythaixoides concolor	f	268	220	245	24	40	sto	plt	2.6
Grey Penduline Tit	Anthoscopus caroli	i	6	51	27	8	13	sho	ovl	4.4
Grey Tit‐Flycatcher	Myioparus plumbeus	i	13	66	58	14	18	sho	hol	2.5
Grey‐backed Camaroptera	Camaroptera brevicaudata	i	11	54	39	12	21	sho	ovl	2.8
Grey‐headed Bush‐Shrike	Malacanotus blanchoti	i	77	114	111	28	32	hoo	cup	2.9
Grey‐rumped Swallow	Pseudhirundo griseopyga	i	10	97	73	5	11	wid	hol	3.3
Groundscraper Thrush	Psophocichla litsitsirupa	i	76	128	69	27	33	sle	cup	2.7
Hadada Ibis	Bostrychia hagedash	i	1,262	353	154	134	68	ben	plt	2.7
Hamerkop	Scopus umbretta	p	422	305	156	82	70	com	ovl	3.3
Helmeted Guineafowl	Numida meleagris	m	1,480	265	171	25	81	sto	gnd	12.5
Jameson's Firefinch	Lagonosticta rhodopareia	g	9	48	41	10	13	con	ovl	3.6
Kori Bustard	Ardeotis kori	m	16,250	678	370	98	206	pro	gnd	2.0
Kurrichane Thrush	Turdus libonyanus	i	60	116	97	22	29	sle	cup	2.9
Lappet‐faced Vulture	Torgos tracheliotus	p	6600	776	351	70	143	hoo	plt	1.0
Laughing Dove	Streptopelia senegalensis	g	103	138	110	16	23	sle	plt	2.0
Lesser Grey Shrike	Lanius minor	i	46	116	89	17	24	hoo	cup	3.5
Lesser Honeyguide	Indicator minor	i	26	88	55	10	14	sto	par	3.0
Lesser Jacana	Microparra capensis	i	41	88	29	17	34	pro	gnd	3.3
Lesser Striped Swallow	Cecropis abyssinica	i	18	112	100	6	10	wid	hol	3.0
Levaillant's Cisticola	Cisticola tinniens	i	12	51	55	11	19	sho	ovl	3.5
Lilac‐breasted Roller	Coracias caudatus	i	106	166	187	33	22	sle	hol	2.8
Little Bee‐eater	Merops pusillus	i	14	80	65	27	8	dec	hol	4.0
Little Grebe	Tachybaptus ruficollis	p	147	101	15	20	27	poi	gnd	3.2
Little Sparrowhawk	Accipiter minullus	p	90	150	117	10	42	hoo	plt	2.0
Lizard Buzzard	Kaupifalco monogrammicus	p	294	226	140	17	53	hoo	plt	1.9
Long‐billed Crombec	Sylvietta rufescens	i	12	61	28	15	19	sli	cup	1.8
Magpie Shrike	Urolestes melanoleucus	i	82	134	282	18	33	hoo	cup	3.3
Malachite Kingfisher	Alcedo cristata	p	15	57	27	34	7	poi	hol	3.7
Marico Flycatcher	Bradornis mariquensis	i	25	85	76	13	21	sho	cup	2.9
Martial Eagle	Polemaetus bellicosus	p	3965	612	288	45	114	hoo	plt	1.0
Meyer's Parrot	Poicephalus meyeri	f	117	152	67	20	17	hoo	hol	2.7
Miombo Double‐collared Sunbird	Cinnyris manoensis	n	9	63	46	24	17	dec	ovl	1.9
Mocking Cliff Chat	Thamnolaea cinnamomeiventris	m	48	112	95	20	29	sli	hol	2.8
Namaqua Dove	Oena capensis	g	40	105	140	14	15	sle	plt	2.0
Natal Spurfowl	Pternistis natalensis	m	458	165	96	19	47	sto	gnd	6.5
Neddicky	Cisticola fulvicapilla	i	8	48	42	11	17	sho	ovl	3.3
Orange‐breasted Bush‐Shrike	Telophorus sulfureopectus	i	27	88	88	16	26	hoo	cup	1.8
Orange‐breasted Waxbill	Amandava subflava	g	8	45	30	9	12	con	ovl	5.0
Pearl‐spotted Owlet	Glaucidium perlatum	p	82	107	76	11	21	hoo	hol	3.0
Pied Crow	Corvus albus	m	519	354	187	59	61	com	cup	4.1
Purple Roller	Coracias naevius	i	168	189	143	41	24	sle	hol	3.3
Quailfinch	Ortygospiza fuscocrissa	m	11	55	28	9	14	con	ovl	4.2
Rattling Cisticola	Cisticola chiniana	i	16	61	60	13	21	sho	ovl	3.1
Red‐billed Buffalo‐Weaver	Bubalornis niger	i	81	119	104	23	30	con	ovl	3.3
Red‐billed Firefinch	Lagonosticta senegala	g	9	48	36	9	12	con	ovl	3.4
Red‐billed Quelea	Quelea quelea	g	19	66	37	14	18	con	ovl	2.0
Red‐billed Teal	Anas erythrorhyncha	m	568	217	81	44	35	dep	gnd	10.0
Red‐breasted Swallow	Cecropis semirufa	i	30	130	118	7	14	wid	hol	3.0
Red‐capped Lark	Calandrella cinerea	i	24	91	62	13	20	con	gnd	2.1
Red‐crested Korhaan	Lophotis ruficrista	m	680	259	133	33	78	pro	gnd	2.0
Red‐eyed Dove	Streptopelia semitorquata	g	235	189	125	22	25	sle	plt	2.0
Red‐faced Mousebird	Urocolius indicus	f	56	96	210	14	18	sto	cup	2.6
Red‐headed Weaver	Anaplectes rubriceps	i	22	80	51	17	19	con	ovl	2.5
Red‐winged Starling	Onychognathus morio	m	139	149	126	28	33	sle	cup	3.1
Retz's Helmetshrike	Prionops retzii	i	48	130	92	24	22	hoo	cup	3.2
Ring‐necked Dove	Streptopelia capicola	g	153	157	101	13	20	sle	plt	2.0
Rosy‐throated Longclaw	Macronyx ameliae	i	33	89	79	15	30	sle	gnd	2.7
Rufous‐naped Lark	Mirafra africana	i	42	95	64	20	29	con	gnd	2.4
Scaly‐feathered Weaver	Sporopipes squamifrons	g	12	57	37	9	15	con	ovl	4.1
Scarlet‐chested Sunbird	Chalcomitra senegalensis	n	13	78	43	29	16	dec	ovl	2.0
Secretary Bird	Sagittarius serpentarius	p	4052	644	700	49	307	hoo	plt	1.9
Senegal Coucal	Centropus senegalensis	p	170	172	205	28	38	sto	ovl	3.5
Shelley's Francolin	Scleroptila shelleyi	m	438	161	79	25	41	sto	gnd	4.8
Shikra	Accipiter badius	p	123	182	137	11	44	hoo	plt	2.5
Southern Black Flycatcher	Melaenornis pammelaina	i	30	104	93	14	23	sho	cup	2.6
Southern Black Tit	Parus niger	i	22	82	71	11	19	sho	hol	3.6
Southern Fiscal	Lanius collaris	i	39	99	106	20	27	hoo	cup	3.5
Southern Grey‐headed Sparrow	Passer diffusus	m	24	81	61	13	18	con	hol	3.3
Southern Masked Weaver	Ploceus velatus	m	26	76	51	16	21	con	ovl	2.6
Southern Red Bishop	Euplectes orix	g	23	71	40	15	21	con	ovl	2.7
Southern White‐crowned Shrike	Eurocephalus anguitimens	i	69	136	108	17	24	hoo	cup	3.3
Southern White‐faced Owl	Ptilopsis granti	p	198	196	93	17	25	hoo	plt	2.4
Southern Yellow‐billed Hornbill	Tockus leucomelas	m	190	198	208	64	38	cas	hol	3.7
Speckled Pigeon	Columba guinea	g	352	226	114	23	34	sle	plt	2.0
Spotted Eagle‐Owl	Bubo africanus	p	666	336	197	39	73	hoo	gnd	2.4
Spotted Thick‐knee	Burhinus capensis	i	453	231	123	37	95	pro	gnd	2.0
Stierling's Wren‐Warbler	Calamonastes stierlingi	i	13	60	45	13	21	sho	ovl	2.5
Striped Kingfisher	Halcyon chelicuti	i	38	83	45	32	11	poi	hol	3.4
Swainson's Spurfowl	Pternistis swainsonii	m	621	183	84	21	56	sto	gnd	6.2
Swallow‐tailed Bee‐eater	Merops hirundineus	i	22	95	103	29	9	dec	hol	3.5
Tawny Eagle	Aquila rapax	p	2,351	523	270	40	86	hoo	plt	1.7
Tawny‐flanked Prinia	Prinia subflava	i	9	49	61	11	20	sho	ovl	3.1
Temminck's Courser	Cursorius temminckii	i	67	124	46	20	40	pro	gnd	1.8
Terrestrial Brownbul	Phyllastrephus terrestris	m	31	90	96	21	25	sli	cup	2.1
Three‐banded Courser	Rhinoptilus cinctus	i	125	163	83	20	72	pro	gnd	2.0
Tropical Boubou	Laniarius aethiopicus	i	50	95	98	23	34	hoo	cup	2.6
Village Indigobird	Vidua chalybeata	g	12	67	36	8	14	con	par	3.0
Village Weaver	Ploceus cucullatus	i	37	85	54	20	21	con	ovl	2.6
Violet‐backed Starling	Cinnyricinclus leucogaster	f	45	107	60	15	20	sle	hol	2.6
Violet‐eared Waxbill	Uraeginthus granatinus	g	12	57	66	11	16	con	ovl	4.5
White‐backed Vulture	Gyps africanus	p	5380	610	258	48	104	hoo	plt	1.0
White‐bellied Sunbird	Cinnyris talatala	n	7	52	33	20	16	dec	ovl	1.9
White‐breasted Cuckoo‐Shrike	Coracina pectoralis	i	58	141	112	19	23	sle	cup	1.5
White‐browed Robin‐Chat	Cossypha heuglini	i	35	98	87	20	30	sli	cup	2.7
White‐browed Scrub Robin	Cercotrichas leucophrys	i	17	68	65	15	24	sli	cup	2.7
White‐browed Sparrow‐Weaver	Plocepasser mahali	m	41	103	63	17	26	con	ovl	2.0
White‐crested Helmetshrike	Prionops plumatus	i	33	107	85	20	21	hoo	cup	3.8
White‐headed Vulture	Trigonoceps occipitalis	p	4700	627	280	51	102	hoo	plt	1.0
White‐necked Raven	Corvus albicollis	p	911	403	182	63	75	com	gnd	3.4
White‐throated Robin‐Chat	Cossypha humeralis	i	21	78	70	16	27	sli	cup	2.7
White‐winged Widowbird	Euplectes albonotatus	g	21	71	61	14	19	con	ovl	2.6
Wire‐tailed Swallow	Hirundo smithii	i	12	107	67	8	7	wid	cup	2.9
Yellow Bishop	Euplectes capensis	g	19	73	55	19	25	con	ovl	2.7
Yellow‐bellied Eremomela	Eremomela icteropygialis	i	7	60	36	11	18	sho	cup	2.3
Yellow‐bellied Greenbul	Chlorocichla flaviventris	m	39	101	96	19	23	sli	cup	2.1
Yellow‐fronted Canary	Crithagra mozambica	m	11	69	41	9	13	con	cup	3.2
Yellow‐fronted Tinkerbird	Pogoniulus chrysoconus	m	13	62	34	13	13	ser	hol	2.5
Yellow‐throated Longclaw	Macronyx croceus	i	48	101	76	18	35	sle	gnd	3.0
Yellow‐throated Petronia	Petronia superciliaris	m	25	91	57	14	19	con	hol	3.1
Zitting Cisticola	Cisticola juncidis	i	9	51	38	10	18	sho	ovl	3.3

Note

The naming convention used is the IOC World Bird List v 7.3.

**TABLE A4:** Species' abundance trends generated by Wild Bird Indices modeling using the multispecies indicator function “msi” in the BRC indicators package (Soldaat et al., 2017)

No. species with >50 individuals	Ranched	Farmed
	61	76
Strong increase	49.2%	46.1%
Moderate increase	14.8%	10.5%
Stable	6.6%	17.1%
Uncertain	21.2%	15.8%
Moderate decline	4.9%	3.9%
Steep decline	3.3%	6.6%

Note

Species included in this analysis were those for which the total number of individuals recorded during the period 2012–2020 in one land‐use area was >50. Trend classifications are as defined in Table A2.
